# Risk of Ischaemic and Non‐Ischaemic Heart Failure in People With Type 2 Diabetes: Observational Study in 1.6 Million People in England

**DOI:** 10.1002/dmrr.70072

**Published:** 2025-07-28

**Authors:** Kajal Panchal, Claire Lawson, Sharmin Shabnam, Kamlesh Khunti, Francesco Zaccardi

**Affiliations:** ^1^ Leicester Real World Evidence Unit Leicester Diabetes Centre University of Leicester Leicester UK; ^2^ Department of Cardiovascular Sciences University of Leicester Leicester UK

**Keywords:** aetiology, diabetes, epidemiology, heart failure, ischaemic heart failure, ischemic heart failure, sex

## Abstract

**Aims/Hypothesis:**

Recent evidence shows decreasing trends for ischaemic heart disease over time in the general population as well as in those with type 2 diabetes. As type 2 diabetes has been associated with an increased risk of both ischaemic and non‐ischaemic heart failure, a greater proportion of people with type 2 diabetes could now be presenting with non‐ischaemic heart failure phenotypes. We aimed to investigate the risk of incident ischaemic and non‐ischaemic heart failure in people with type 2 diabetes.

**Methods:**

We used the Clinical Practice Research Datalink primary care data, linked to hospital and mortality records, to identify newly diagnosed adults with type 2 diabetes between 2000 and 2021, who were matched to up to four people without diabetes by sex, year of birth, and general practice. Ischaemic heart failure was defined as incident heart failure at or following an ischaemic heart disease event; non‐ischaemic HF was defined as incident heart failure in the absence of prevalent ischaemic heart disease. We used Poisson and Royston‐Parmar models to estimate, respectively, the incidence rates and the hazard ratios (adjusted for sociodemographic and clinical confounders) for ischaemic and non‐ischaemic heart failure, comparing people with type 2 diabetes to those without diabetes.

**Results:**

In a cohort of 1,621,090 people (mean age, 60.1 years; 52.8% women; 532,185 with type 2 diabetes), during a median follow‐up of 5.8 (interquartile range: 2.6–10.3) years, a heart failure event occurred in 20,016 (3.8%) people with type 2 diabetes (ischaemic: 5046; non‐ischaemic: 14,970) and in 29,835 (2.7%) without diabetes (7001 and 22,834, respectively). Age‐standardised rates were higher for non‐ischaemic (3.18 [95% CI: 3.09–3.27] vs. 2.08 [2.03–2.12] per 1000 person‐years in men with type 2 diabetes vs. without diabetes; and 2.47 [2.39–2.54] vs. 1.57 [1.53–1.61], respectively, in women) than ischaemic (corresponding estimates: 1.57 [1.51–1.63] vs. 0.95 [0.92–0.98] and 0.80 [0.76–0.84] vs. 0.46 [0.44–0.48]) heart failure. Comparing people with type 2 diabetes versus those without diabetes, the hazard ratios were larger for ischaemic (adjusted hazard ratio: 1.36 [1.28–1.45] and 1.30 [1.20–1.42] in men and women, respectively) than non‐ischaemic (1.12 [1.07–1.16] and 1.10 [1.06–1.14], respectively) heart failure.

**Conclusions/Interpretations:**

The higher rates of non‐ischaemic heart failure highlight the need for early prevention before ischaemic heart disease develops, regardless of type 2 diabetes. Meanwhile, the greater excess risk of ischaemic heart failure in those with type 2 diabetes suggests suboptimal post‐ischaemic prevention in this group.

## Graphical abstract

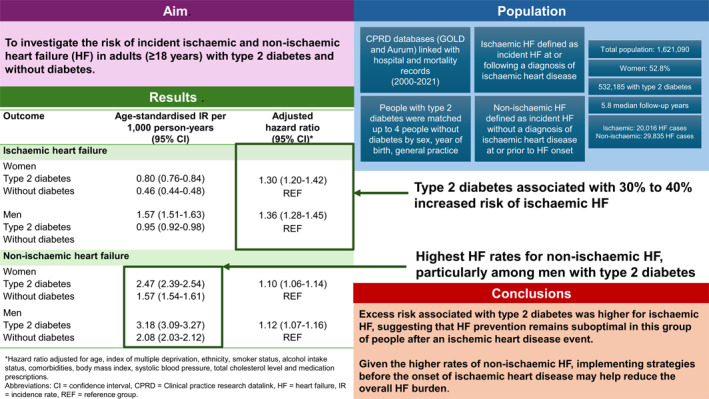

## 1 Introduction

Previous observational studies have demonstrated an increased risk of heart failure in people with type 2 diabetes compared with those without diabetes [1]. This association is often a consequence of atherothrombotic ischaemic heart diseases (i.e., coronary artery disease or myocardial infarction, MI) associated with type 2 diabetes, leading to ischaemic heart failure [2]. However, some cases of heart failure may develop without a prior diagnosis of ischaemic heart disease, that is, non‐ischaemic heart failure. In such instances, hyperglycaemia and insulin resistance associated with diabetes exacerbate myocardial oxidative stress and inflammation, which could result in direct myocardial damage and a higher risk of non‐ischaemic heart failure [3, 4].

Recent evidence suggests declining trends in the incidence of ischaemic heart disease and MI in people with and without diabetes [5, 6]. As such, compared to previous years, a higher proportion of people could now be presenting with non‐ischaemic rather than ischaemic heart failure. However, whether and to what extent such trends have altered the aetiology of heart failure remains unclear. Furthermore, previous observational studies have reported an increased risk of incident heart failure in people with type 2 diabetes from the UK [2, 7, 8, 9], as well as other countries [10]. However, to the author's knowledge, no known studies have comprehensively explored whether and to what extent this association differs according to HF aetiology (ischaemic and non‐ischaemic) in people with type 2 diabetes. This information is necessary to improve our knowledge of the epidemiological heart failure patterns, improve heart failure prognosis and guide more tailored strategies to reduce the total burden of heart failure in people with and without type 2 diabetes [11].

In this study, we aimed to investigate the association between type 2 diabetes and incident ischaemic and non‐ischaemic heart failure using a large UK primary care dataset linked to hospital and mortality records.

## 2 Methods

### 2.1 Data Sources

This study used data from the Clinical Practice Research Datalink (CPRD) database linked to the Hospital Episode Statistics Admitted Patient Care (HES APC) data and to the Office for National Statistics (ONS) death registration data in England. CPRD is an electronic health record database that captures information on primary care patient demographic, clinical, laboratory, and medical records (https://www.cprd.com/), and is representative of the UK population with respect to age, sex, and ethnicity [12, 13]. All people from CPRD were included if they had available linkages with HES APC and ONS data. This study followed a pre‐registered protocol approved by the Independent Scientific Advisory Committee of the Medicines and Healthcare products Regulatory Authority (protocol number: 21_000355) and was conducted in accordance with the RECORD guidelines (checklist provided in Supporting Information S1). All codes used to define the population, characteristics, and outcomes are available on GitHub (link: https://github.com/KajalPanchalProjects/Codelists‐and‐Statacode1).

### 2.2 Study Population

This is an observational, retrospective cohort study including all adults (≥ 18 years) in CPRD GOLD and Aurum with a first recorded diagnosis of type 2 diabetes (main exposure) between 1 January 2000 and 29 March 2021; the date of the first recorded diagnosis was the index date. People with type 2 diabetes were matched at random with up to four individuals without type 2 diabetes by sex, year of birth, and general practice without replacement. If there were fewer than four eligible matched individuals, all available controls were matched; if there were no suitable matched individuals identified, the case was considered unmatched (Supporting Information S1: Table S1). This matching approach allowed us to maintain a large sample size, thus enhancing statistical power while enabling detailed population stratification by sex and heart failure aetiology. All people included had been registered for a minimum of 12 months prior to their index date (or equivalent matched index date for those without diabetes) and were of acceptable research standards. People were excluded if their date of death from ONS, transfer out date in CPRD, last collection date in CPRD, last linkage date from HES, or the study end date (21 March 2019) was at or prior to the index date. People were excluded if they had prevalent cardiovascular disease (ischaemic heart disease [coronary heart disease, angina, MI, coronary artery bypass graft or percutaneous coronary intervention], peripheral vascular disease, or stroke) or the primary outcome of interest (heart failure) prior to or at the index date in CPRD GOLD, Aurum, or HES. A detailed flowchart of the study population is reported in Supporting Information S1: Figures S1 and S2.

### 2.3 Main Exposure and Covariates

The primary exposure of interest was type 2 diabetes, defined as the first recorded clinical code in CPRD. Covariates included age (years) at index date, sex, ethnicity (White, Black, South Asian, Mixed/Other, Unknown—defined using HES), and deprivation (measured in quintiles of the Index of Multiple Deprivation, a proxy for individual‐level socio‐economic status: IMD first, least deprived; fifth, most deprived) [14]. We extracted information on the following cardiovascular risk factors: smoking status (ever‐smoker, never‐smoker), alcohol consumption (current, ex, never), comorbidities (anaemia, asthma, atrial fibrillation, cancer, chronic kidney disease, chronic liver disease, chronic obstructive pulmonary disease, dementia, depression, hypertension, osteoarthritis, rheumatoid arthritis, and thyroid disorders defined in CPRD or HES), medication prescriptions (antiplatelet, antihypertensive, and lipid‐lowering medications, digoxin), body mass index (BMI), systolic blood pressure, and total cholesterol. All cardiovascular risk factors were extracted from the latest available records before or at the index date.

### 2.4 Outcomes

The primary outcomes were ischaemic heart failure and non‐ischaemic heart failure, defined as the first diagnosis code in CPRD GOLD, CPRD Aurum, HES (primary cause of hospitalisation), or ONS (primary underlying cause of death) after the index date. Ischaemic heart failure events were defined as heart failure episodes at or following a diagnosis of ischaemic heart disease (coronary heart disease, angina, MI, coronary artery bypass graft or percutaneous coronary intervention) since the index date, while non‐ischaemic heart failure events were defined as heart failure episodes without a diagnosis of ischaemic heart disease since the index date. Secondary outcome included total heart failure (i.e., ischaemic, or non‐ischaemic heart failure). The ONS linkage end date for this study was 29 March 2021.

### 2.5 Statistical Analysis

We summarised the sex‐specific baseline characteristics at index date by exposure status (all individuals with type 2 diabetes and matched individuals without diabetes) as number (proportion) for categorical data and mean (SD) for continuous data. We used Poisson regression models to estimate sex‐stratified crude and age‐standardised rates (at the mean age of the total population, 60 years old) for all outcomes (ischaemic heart failure, non‐ischaemic heart failure, total heart failure) in people with type 2 diabetes and without diabetes. Poisson models were also used to estimate sex‐stratified crude and age‐adjusted incidence rate ratios (IRRs) for all outcomes comparing people with type 2 diabetes to those without diabetes.

For all outcomes and each sex, we estimated the hazard ratios, comparing people with type 2 diabetes to those without diabetes, using Royston‐Parmar parametric models with the following adjustments: Model 1 = unadjusted; Model 2 = adjusted for age; Model 3 = Model 2 + IMD, ethnicity, smoking, alcohol intake; Model 4 = Model 3 + comorbidities (anaemia, asthma, atrial fibrillation, cancer, chronic kidney disease, chronic liver disease, chronic obstructive pulmonary disease, dementia, depression, hypertension, osteoarthritis, rheumatoid arthritis, and thyroid disorders), BMI, systolic blood pressure, total cholesterol level; Model 5 = Model 4 + medications. In analyses investigating ischaemic heart failure, we censored those who had a diagnosis of non‐ischaemic heart failure and vice versa. All analyses were complete‐case (Supporting Information S1: Table S2) and were performed using Python 3.8, Stata/BE 17.0 and 18.0.

### 2.6 Sensitivity Analyses

To assess the robustness of the outcome definition, we repeated the analyses defining ischaemic heart failure and non‐ischaemic heart failure exclusively as heart failure hospitalisation (using HES, primary cause of hospitalisation) or heart failure death (using ONS, primary underlying cause of death), corresponding to the most severe cases of heart failure. We also repeated the analyses excluding people with prevalent peripheral vascular disease and stroke prior to or at the index date but including those with prevalent ischaemic heart disease (coronary heart disease, angina, MI, coronary artery bypass graft or percutaneous coronary intervention) prior to or at the index date; this allowed us to define ischaemic heart failure as an incident heart failure episode with prior diagnosis of ischaemic heart disease any time before the heart failure event.

## 3 Results

### 3.1 Cohort Characteristics

The final cohort included 1,621,090 people: 855,342 women (274,743 with type 2 diabetes and 580,599 without diabetes) and 765,748 men (257,442 and 508,306, respectively). Table 1 shows the cohort characteristics stratified by diabetes and sex. In both men and women, among those with type 2 diabetes versus those without diabetes, there were fewer people of White ethnicity (78% vs. 87% in women; 79% vs. 87% in men) and in the least deprived group (16% vs. 20% and 18% vs. 21%, respectively). BMI was higher in people with type 2 diabetes (32.3 vs. 26.7 kg/m^2^ in women; 31.1 vs. 26.9 kg/m^2^ in men) as well as systolic blood pressure (136 vs. 132 mmHg and 139 vs. 134 mmHg, respectively). Overall, comorbidities were more prevalent in people with type 2 diabetes versus without diabetes. The cohort characteristics stratified by incident heart failure (ischaemic and non‐ischaemic) are reported in Supporting Information S1: Tables S3 (women) and S4 (men).

**TABLE 1:** Sex‐stratified baseline characteristics.

	Women		Men	
	People with type 2 diabetes	People without diabetes	People with type 2 diabetes	People without diabetes
	*n* = 274,743	*n* = 580,599	*n* = 257,442	*n* = 508,306
Age (years)	58.74 (15.33)	60.87 (13.96)	58.77 (13.10)	60.73 (11.75)
Ethnicity				
White	214,248 (77.98)	507,119 (87.34)	203,889 (79.20)	443,707 (87.29)
South Asian	21,419 (7.80)	16,419 (2.83)	16,325 (6.34)	11,962 (2.35)
Black	15,915 (5.79)	14,807 (2.55)	11,443 (4.44)	10,435 (2.05)
Mixed/Other	13,881 (5.05)	15,740 (2.71)	13,294 (5.16)	12,836 (2.53)
Unknown	9280 (3.38)	26,514 (4.57)	12,491 (4.85)	29,366 (5.78)
IMD quintile				
1 (least deprived)	44,263 (16.11)	118,030 (20.33)	45,192 (17.55)	109,113 (21.47)
2	49,091 (17.87)	122,670 (21.13)	48,857 (18.98)	110,317 (21.70)
3	53,188 (19.36)	115,038 (19.81)	50,462 (19.60)	101,499 (19.97)
4	61,055 (22.22)	113,742 (19.59)	55,371 (21.51)	96,107 (18.91)
5 (most deprived)	67,146 (24.44)	111,119 (19.14)	57,560 (22.36)	91,270 (17.96)
Smoking status				
Ever‐smoker	116,549 (42.42)	243,454 (41.93)	147,875 (57.44)	275,568 (54.21)
Non‐smoker	158,194 (57.58)	337,145 (58.07)	109,567 (42.56)	232,738 (45.79)
Alcohol intake status				
Current	197,897 (82.28)	424,564 (85.62)	201,634 (90.21)	396,377 (92.45)
Ex‐drinker	2415 (1.00)	3581 (0.72)	2666 (1.19)	3508 (0.82)
Never	40,190 (16.71)	67,734 (13.66)	19,221 (8.60)	28,880 (6.74)
BMI (kg/m^2^)	32.33 ± 7.70	26.74 ± 5.55	31.10 ± 6.18	26.85 ± 4.40
Systolic BP (mmHg)	136.17 ± 18.21	131.77 ± 17.35	138.76 ± 16.91	134.21 ± 15.68
Total cholesterol (mmol/L)	4.80 ± 1.53	4.87 ± 1.45	4.72 ± 1.60	4.69 ± 1.32
Comorbidities				
Anaemia	31,995 (11.65)	48,346 (8.33)	10,392 (4.04)	16,372 (3.22)
Asthma	57,017 (20.75)	86,336 (14.87)	37,436 (14.54)	62,454 (12.29)
Atrial fibrillation	8341 (3.04)	11,383 (1.96)	9491 (3.69)	14,722 (2.90)
Cancer	35,471 (12.91)	76,760 (13.22)	25,881 (10.05)	53,239 (10.47)
Chronic kidney disease	31,916 (11.62)	57,537 (9.91)	18,943 (7.36)	30,599 (6.02)
Chronic liver disease	6484 (2.36)	5620 (0.97)	6920 (2.69)	6115 (1.20)
COPD	13,949 (5.08)	23,148 (3.99)	14,291 (5.55)	24,029 (4.73)
Dementia	3298 (1.20)	6857 (1.18)	1979 (0.77)	4322 (0.85)
Depression	79,017 (28.76)	137,948 (23.76)	42,976 (16.69)	70,497 (13.87)
Hypertension	130,061 (47.34)	169,706 (29.23)	117,585 (45.67)	141,802 (27.90)
Osteoarthritis	53,945 (19.63)	90,329 (15.56)	36,934 (14.35)	61,259 (12.05)
Rheumatoid arthritis	6390 (2.33)	12,574 (2.17)	2868 (1.11)	5316 (1.05)
Thyroid disorders	39,847 (14.50)	60,196 (10.37)	9292 (3.61)	12,423 (2.44)
Medication prescription				
Antihypertensive drugs	170,870 (62.19)	260,796 (44.92)	144,202 (56.01)	200,707 (39.49)
Antiplatelet drugs	45,454 (16.54)	65,156 (11.22)	41,860 (16.26)	56,270 (11.07)
Digoxin	4075 (1.48)	3831 (0.66)	3419 (1.33)	4069 (0.80)
Lipid lowering drugs	82,572 (30.05)	90,363 (15.56)	90,523 (35.16)	93,961 (18.49)

*Note:* All categorical variables are reported as number (proportion, %) and continuous variables as mean (standard deviation). Cohort of people without missing data on ethnicity, IMD, systolic BP, smoking status, BMI (Supporting Information S1: Figures S1 and S2). Missing data for total cholesterol and alcohol intake are reported in Supporting Information S1: Table S1.

Abbreviations: BMI = body mass index, BP = blood pressure, COPD = chronic obstructive pulmonary disease, IMD = index of multiple deprivation, *n* = number of people.

### 3.2 Incidence of Heart Failure

During a median follow‐up of 5.8 (interquartile range [IQR]: 2.6–10.3) years and 11,153,061 person‐years, 49,851 (3.1%) people experienced a heart failure event: 9496 and 10,520 women and men, respectively, with type 2 diabetes; and 14,586 and 15,249 without diabetes (Table 2). The crude (5.44 and 6.23 per 1000 person‐years in women and men, respectively) and age‐standardised (3.28 and 4.81) incidence rates in people with type 2 diabetes were higher than in those without diabetes (3.55 and 4.22; 2.04 and 3.06): these estimates translated to age‐adjusted IRRs of 1.61 (95% CI: 1.57–1.65) in women and 1.57 (1.53–1.61) in men comparing people with type 2 diabetes to those without diabetes. In the maximally adjusted model (Model 5) for the same comparison, the HRs were 1.13 (95% CI: 1.10–1.17) in women and 1.18 (1.14–1.22) in men (Table 3).

**TABLE 2:** Sex‐stratified rates of incident heart failure.

Outcome, sex, group	Event (*n*)	Crude IR per 1000 person‐years (95% CI)	Age‐standardised IR per 1000 person‐years (95% CI)	Crude IRR (95% CI)	Age‐adjusted IRR (95% CI)
Heart failure					
Women					
With type 2 diabetes	9496	5.44 (5.33–5.54)	3.28 (3.20–3.37)	1.53 (1.49–1.57)	1.61 (1.57–1.65)
Without diabetes	14,586	3.55 (3.50–3.61)	2.04 (2.00–2.08)	REF	REF
Men					
With type 2 diabetes	10,520	6.23 (6.11–6.34)	4.81 (4.70–4.91)	1.47 (1.44–1.51)	1.57 (1.53–1.61)
Without diabetes	15,249	4.22 (4.16–4.29)	3.06 (3.00–3.11)	REF	REF
Ischaemic heart failure					
Women					
With type 2 diabetes	1967	1.11 (1.06–1.16)	0.80 (0.76–0.84)	1.66 (1.57–1.76)	1.75 (1.66–1.86)
Without diabetes	2774	0.67 (0.65–0.70)	0.46 (0.44–0.48)	REF	REF
Men					
With type 2 diabetes	3079	1.80 (1.74–1.86)	1.57 (1.51–1.63)	1.55 (1.48–1.62)	1.65 (1.57–1.73)
Without diabetes	4227	1.16 (1.13–1.20)	0.95 (0.92–0.98)	REF	REF
Non‐ischaemic heart failure					
Women					
With type 2 diabetes	7529	4.30 (4.20–4.39)	2.47 (2.39–2.54)	1.50 (1.45–1.54)	1.57 (1.52–1.61)
Without diabetes	11,812	2.87 (2.82–2.92)	1.57 (1.54–1.61)	REF	REF
Men					
With type 2 diabetes	7441	4.38 (4.28–4.48)	3.18 (3.09–3.27)	1.44 (1.40–1.48)	1.53 (1.49–1.58)
Without diabetes	11,022	3.04 (2.99–3.10)	2.08 (2.03–2.12)	REF	REF

*Note:* Age‐standardised rates were estimated at mean age of the total population at 60 years old.

Abbreviations: CI = confidence interval, IR = incidence rate, IRR = incidence rate ratio, *n* = number of people, REF = reference group.

**TABLE 3:** Sex‐stratified hazard ratios of incident heart failure.

Outcome, sex	Model 1		Model 2		Model 3		Model 4		Model 5	
	Events/*N*	HR (95% CI)	Events/*N*	HR (95% CI)	Events/*N*	HR (95% CI)	Events/*N*	HR (95% CI)	Events/*N*	HR (95% CI)
Heart failure										
Women	24,082/855,342	1.60 (1.55–1.64)	24,082/855,342	1.71 (1.67–1.76)	21,438/736,381	1.65(1.60–1.70)	14,674/517,138	1.15 (1.11–1.19)	14,674/517,138	1.13 (1.10–1.17)
Men	25,769/765,748	1.52 (1.48–1.56)	25,769/765,748	1.65 (1.61–1.69)	22,745/652,286	1.60 (1.56–1.64)	15,948/478,750	1.18 (1.14–1.22)	15,948/478,750	1.18 (1.14–1.22)
Ischaemic heart failure										
Women	4741/855,342	1.77 (1.67–1.87)	4741/855,342	1.90 (1.80–2.02)	4191/736,381	1.81 (1.70–1.93)	2750/517,138	1.33 (1.23–1.44)	2750/517,138	1.30 (1.20–1.42)
Men	7306/765,748	1.62 (1.55–1.70)	7306/765,748	1.75 (1.67–1.83)	6351/652,286	1.67 (1.59–1.75)	4346/478,750	1.37 (1.28–1.46)	4346/478,750	1.36 (1.28–1.45)
Non‐ischaemic heart failure										
Women	19,341/855,342	1.55 (1.50–1.59)	19,341/855,342	1.65 (1.61–1.70)	17,247/736,381	1.60 (1.55–1.65)	11,924/517,138	1.11 (1.06–1.15)	11,924/517,138	1.10 (1.06–1.14)
Men	18,463/765,748	1.47 (1.43–1.52)	18,463/765,748	1.59 (1.54–1.64)	16,394/652,286	1.56 (1.51–1.61)	11,602/478,750	1.11 (1.07–1.16)	11,602/478,750	1.12 (1.07–1.16)

*Note:* Reference group for women: women without diabetes; reference group for men: men without diabetes. Model 1) Unadjusted. Model 2) Adjusted for age. Model 3) Adjusted for age, index of multiple deprivation, ethnicity, smoking, and alcohol intake status. Model 4) Adjusted for age, index of multiple deprivation, ethnicity, smoking, alcohol intake status and comorbidities (anaemia, asthma, atrial fibrillation, cancer, chronic kidney disease, chronic liver disease, chronic obstructive pulmonary disease, dementia, depression, hypertension, osteoarthritis, rheumatoid arthritis, thyroid disorders), body mass index, systolic blood pressure, total cholesterol level. Model 5) Adjusted for age, index of multiple deprivation, ethnicity, smoker status, alcohol intake status, comorbidities (anaemia, asthma, atrial fibrillation, cancer, chronic kidney disease, chronic liver disease, chronic obstructive pulmonary disease, dementia, depression, hypertension, osteoarthritis, rheumatoid arthritis, thyroid disorders), body mass index, systolic blood pressure, total cholesterol level and prescriptions (antihypertensive medications, antiplatelets medications, digoxin, and lipid‐lowering medications).

Abbreviations: CI = confidence interval, HR = hazard ratio, *N* = number of people.

In people with type 2 diabetes, 1967 women and 3079 men experienced an ischaemic heart failure event, corresponding to 20.7% and 29.3%, respectively, of all heart failure events; equivalent estimates in people without diabetes were 2774 in women, 4227 in men, and 19.0% and 27.7% (Table 2). The crude (1.11 and 1.80 per 1000 person‐years in women and men, respectively) and age‐standardised (0.80 and 1.57) incidence rates in people with type 2 diabetes were higher than in those without diabetes (0.67 and 1.16; 0.46 and 0.95). These rates equated to crude IRRs of 1.66 (95% CI: 1.57–1.76) and 1.55 (1.48–1.62) in women and men, respectively, comparing people with type 2 diabetes versus those without diabetes; and in higher age‐adjusted IRRs of 1.75 (1.66–1.86) and 1.65 (1.57–1.73), respectively (Table 2). In women, the HRs for type 2 diabetes (vs. without diabetes) ranged from a maximum of 1.90 (95% CI: 1.80–2.02) in a model adjusted for age (Model 2) to a minimum of 1.30 (1.20–1.42) in the maximally adjusted model (Model 5); corresponding estimates in men ranged from 1.75 (1.67–1.83) to 1.36 (1.28–1.45) (Table 3).

In women, 7529 with type 2 diabetes and 11,812 without diabetes experienced a non‐ischaemic heart failure event, corresponding to 79.3% and 81.0%, respectively, of all heart failure events; equivalent estimates in men were 7441 in people with type 2 diabetes, 11,022 in those without diabetes, and 70.7% and 72.3% (Table 2). The crude (4.30 and 4.38 per 1000 person‐years in women and men, respectively) and age‐standardised (2.47 and 3.18) incidence rates in people with type 2 diabetes were higher than in those without diabetes (2.87 and 3.04; 1.57 and 2.08), corresponding to crude IRRs of 1.50 (95% CI: 1.45–1.54) and 1.44 (1.40–1.48) in women and men, respectively, comparing people with type 2 diabetes versus without diabetes and to age‐adjusted IRRs of 1.57 (1.52–1.61) and 1.53 (1.49–1.58), respectively (Table 2). The HRs for type 2 diabetes (vs. without diabetes) ranged from a maximum of 1.65 (95% CI: 1.61–1.70) in a model adjusted for age (Model 2) to a minimum of 1.10 (1.06–1.14) in the maximally adjusted model (Model 5) in women and, correspondingly, from 1.59 (1.54–1.64) to 1.12 (1.07–1.16) in men (Table 3).

### 3.3 Sensitivity Analyses

When defining heart failure as heart failure hospitalisation or heart failure death, the pattern of results was consistent with that observed in the main findings but with moderately higher age‐adjusted IRRs (Supporting Information S1: Table S5) and HRs (Supporting Information S1: Table S6). Findings were also consistent when including people with prevalent ischaemic heart disease at the study baseline: for ischaemic and non‐ischaemic heart failure, but age adjusted IRRs were slightly higher or lower, respectively (Supporting Information S1: Table S7); and HRs slightly lower or very similar HRs, respectively (Supporting Information S1: Table S8).

## 4 Discussion

In this study, we investigated the incidence of ischaemic and non‐ischaemic heart failure in a cohort of primary care patients with and without type 2 diabetes in England. In both men and women, we found higher rates for non‐ischaemic than ischaemic heart failure, irrespective of diabetes: the lowest age‐standardised rates of non‐ischaemic heart failure—observed in women without type 2 diabetes (1.6 per 1000 person‐years)—was virtually identical to the highest rates of ischaemic heart failure—observed in men with type 2 diabetes. However, in both sexes, type 2 diabetes was associated with an excess in rates higher for ischaemic (HR in men: 1.4; women: 1.3) than non‐ischaemic (men: 1.1; women: 1.1) heart failure. Therefore, our results highlight a dichotomy: while crude and age‐standardised rates were higher for non‐ischaemic heart failure, the relative risk associated with type 2 diabetes was greater for the ischaemic phenotype. These findings suggest that future intervention studies should assess whether reducing the total burden of heart failure could achieve greater population‐wide effects when implemented prior to the diagnosis of ischaemic heart disease. Additionally, it would be valuable to investigate how improving the identification, prevention, and control of atherothrombotic risk factors in people with type 2 diabetes, as well as their management following an ischaemic heart disease event, could reduce the disparities in heart failure risk, particularly for ischaemic heart failure.

Our results showing higher rates of incident non‐ischaemic than ischaemic heart failure in men and women are in line with a 2022 UK study reporting increasing trends of prevalent cardiomyopathies between 2010 and 2018 [15], possibly resulting in greater rates of non‐ischaemic heart failure. They are also consistent with other recent UK studies showing decreasing trends in people with and without diabetes of (i) the overall incidence of ischaemic heart disease [16, 17], and (ii) the incidence of MI [6]. There were several possible reasons accounting for the greater rates of non‐ischaemic heart failure; notably, a 41.8% increase in the prescriptions of cardiovascular disease medications has been reported in England and Wales between 2004 and 2019, a pattern which has been associated with lower rates of ischaemic heart disease hospitalisations [17]. Other possible reasons include improvements in hypertension awareness, management, and treatment over time in the UK [18, 19, 20, 21] as well as reducing trends in smoking prevalence [22].

The differences between men and women in the age‐standardised IRs were similar: 0.8 and 0.7 per 1000 person‐years for ischaemic and non‐ischaemic heart failure, respectively, in people with type 2 diabetes; and 0.5 per 1000 person‐years for both ischaemic and non‐ischaemic heart failure, in people without diabetes. Recent UK studies have reported greater rates of total incident heart failure in men than women with and without type 2 diabetes [7, 16], alongside others showing higher rates of ischaemic heart diseases [6, 16] and prevalence of non‐ischaemic heart diseases (i.e., idiopathic [23], dilated [15], or hypertrophic cardiomyopathy [15]) in men than women. While the cardioprotective effects of oestrogen could be one potential mechanism underpinning the lower risk of cardiovascular diseases in women [24, 25, 26], our findings might also reflect differences in comorbidity profiles between men and women [27] or the under‐, late‐, or misdiagnosis of ischaemic heart disease in women [28]. Moreover, the greater rates of non‐ischaemic heart failure observed in men versus women with type 2 diabetes could be related to greater ectopic (i.e., visceral fat accumulation) found in men than women [29], with the resulting amplified insulin resistance accelerating the risk of non‐ischaemic HF via direct myocardial damage [3, 4, 30]. However, future research investigating the sex‐specific underlying mechanisms for these associations is needed to confirm this.

In both men and women, accounting for potential confounders, the HRs associated with type 2 diabetes were higher for ischaemic than non‐ischaemic heart failure: 1.36 versus 1.12 in men and 1.30 versus 1.10 in women. To our knowledge, no previous studies have specifically explored the differential association between type 2 diabetes and the phenotypes of heart failure in the same population, limiting comparisons with previous literature. A 2020 small study included people with ischaemic heart disease (of which 96 had diabetes) and reported that diabetes was associated with a two‐fold adjusted increased hazard rate of heart failure (HR: 2.04, 95% CI: 1.32–3.14) compared to those without diabetes [31]. However, this study had a small sample size (*n* = 306) and did not objectively define people with type 2 diabetes, provide sex‐specific estimates, or comprehensively adjust for well‐known confounders (i.e., chronic kidney disease, BMI or ethnicity). In addition, this was a single‐centre study using hospital data, hindering representativeness to the general population. Conversely, our study is based on a very large and more homogenous single population, with analyses progressively adjusted for several clinically relevant confounders to reduce the risk of biased estimates. Given the difference in the populations, risk factor distributions, and healthcare systems, it is difficult to (indirectly) compare whether the association of type 2 diabetes with heart failure differs by heart failure phenotype.

Further strengths of our study include the use of CPRD data, a validated source of real‐world data, representative of the UK population in terms of age and sex [12, 13]; moreover, the inclusion of non‐overlapping data across CPRD GOLD and Aurum allowed us to capture a large population (∼1.6 million) and number of heart failure cases (∼20,000) during a long follow‐up (∼11 million total person‐years). This also permitted population stratification by sex to better understand the associations between type 2 diabetes and heart failure aetiology and inform policy with greater detail. Additionally, our analysis was performed in a primary cardiovascular prevention population; we defined heart failure using an incident study design and excluded people with cardiovascular disease (stroke, peripheral vascular disease, ischaemic heart disease, heart failure) at cohort baseline. This approach minimises residual confounding and allows us to better understand the temporal association between type 2 diabetes and incident ischaemic versus non‐ischaemic heart failure. Furthermore, we used routinely collected health records to define heart failure based on primary care, hospital and morality data, reflecting real‐world evidence and improving the generalisability of our findings.

However, there are also some limitations, whereby using routinely collected health records might have led to measurement errors for some covariates and heart failure outcomes as these data are not primarily collected for research. Additionally, we defined people with type 2 diabetes using clinical codes alone when diabetes algorithms incorporating clinical‐demographic factors, laboratory tests, and medications have been proposed [32]; still there is evidence of high accuracy in identifying cases of type 2 diabetes using clinical codes alone versus algorithms [33]. We acknowledge the potential of misclassification bias due to diagnostic delays, where undiagnosed ischaemic heart disease at the date of heart failure diagnosis might have been identified at a later date. This could have led to an underreporting of ischaemic heart failure cases and an overestimation of non‐ischaemic heart failure cases in the current study. Furthermore, given the large sample size, it was computationally very intensive to impute missing data; therefore, we used a complete case analysis. Moreover, since echocardiography data were unavailable, it was not possible to differentiate between heart failure with or without reduced ejection fraction. In addition, a few confounding variables (i.e., body fat distribution, physical activity, dietary factors) were unavailable in the CPRD and HES databases and could not be included in the models. Lastly, we recognise that there are a range of underlying causes of non‐ischaemic heart failure; further research investigating this phenotype in greater detail would be useful to enhance our understanding.

In conclusion, our findings showed greater rates of non‐ischaemic than ischaemic heart failure in both people with and without type 2 diabetes; however, the excess risk associated with type 2 diabetes was larger for ischaemic than non‐ischaemic heart failure. Future studies should investigate whether effective strategies implemented before the onset of ischaemic heart disease could help reduce the overall burden of heart failure. Additionally, it is crucial to explore how improving the management of individuals with type 2 diabetes following an ischaemic heart disease event might help narrow the risk gap in ischaemic heart failure.

## Author Contributions

K.P. contributed to the conception and design of work, data cleaning, data analysis, validation, and interpretation of results, including drafting the article and revising the draft for important intellectual content. C.L. and F.Z. contributed to the conception and design of work, supervision, data cleaning, data analysis, validation, and interpretation of the results, including revising the draft for important intellectual content. S.S. contributed to the data cleaning and analysis, interpretation of the results and revising the draft for important intellectual content. K.K. contributed to the conception and design of the work, interpretation of the results and revising the draft for important intellectual content.

## Conflicts of Interest

All authors have completed the ICMJE uniform disclosure form at www.icmje.org/coi_disclosure.pdf. K.P. PhD was supported by the NIHR ARC EM. F.Z. and C.L. are supported by the NIHR ARC EM and NIHR Leicester Biomedical Research Centre. F.Z. received consultation fees from Daiichi Sankyo. K.K. has acted as a consultant, speaker or received grants for investigator‐initiated studies for Astra Zeneca, Bayer, Novartis, Novo Nordisk, Sanofi‐Aventis, Lilly and Merck Sharp & Dohme, Boehringer Ingelheim, Oramed Pharmaceuticals and Applied Therapeutics. S.S. reports no conflicts of interest.

## Peer Review

The peer review history for this article is available at https://www.webofscience.com/api/gateway/wos/peer-review/10.1002/dmrr.70072.

## Supporting information

Supporting Information S1

## Data Availability

Data is available upon approval from CPRD only.

## References

[1] S. M. Dunlay , M. M. Givertz , D. Aguilar , et al., “Type 2 Diabetes Mellitus and Heart Failure: A Scientific Statement From the American Heart Association and the Heart Failure Society of America: This Statement Does Not Represent an Update of the 2017 ACC/AHA/HFSA Heart Failure Guideline Update,” Circulation 140, no. 7 (August 13, 2019): e294–e324. [Internet]. [cited 2024 May 16], 10.1161/CIR.0000000000000691. 31167558

[2] A. D. Shah , C. Langenberg , E. Rapsomaniki , et al., “Type 2 Diabetes and Incidence of Cardiovascular Diseases: A Cohort Study in 1·9 Million People,” Lancet Diabetes & Endocrinology 3, no. 2 (February 2015): 105–113, 10.1016/S2213-8587(14)70219-0. 25466521 PMC4303913

[3] H. Bugger and E. D. Abel , “Molecular Mechanisms of Diabetic Cardiomyopathy,” Diabetologia 57, no. 4 (April 2014): 660–671, 10.1007/s00125-014-3171-6. 24477973 PMC3969857

[4] M. M. Y. Lee , J. J. V. McMurray , A. Lorenzo‐Almorós , et al., “Diabetic Cardiomyopathy,” Heart 105, no. 4 (February 2019): 337–345, 10.1136/heartjnl-2016-310342. 30337334

[5] E. W. Gregg , N. Sattar , and M. K. Ali , “The Changing Face of Diabetes Complications,” Lancet Diabetes & Endocrinology 4, no. 6 (June 2016): 537–547, 10.1016/S2213-8587(16)30010-9. 27156051

[6] S. H. Read , C. M. Fischbacher , H. M. Colhoun , et al., “Trends in Incidence and Case Fatality of Acute Myocardial Infarction, Angina and Coronary Revascularisation in People With and Without Type 2 Diabetes in Scotland Between 2006 and 2015,” Diabetologia 62, no. 3 (March 2019): 418–425, 10.1007/s00125-018-4796-7. 30656362 PMC7019674

[7] D. A. McAllister , S. H. Read , J. Kerssens , et al., “Incidence of Hospitalization for Heart Failure and Case‐Fatality Among 3.25 Million People With and Without Diabetes Mellitus,” Circulation 138, no. 24 (December 11, 2018): 2774–2786, 10.1161/CIRCULATIONAHA.118.034986. 29950404 PMC6287897

[8] S. Chadalavada , M. T. Jensen , N. Aung , et al., “Women With Diabetes Are at Increased Relative Risk of Heart Failure Compared to Men: Insights From UK Biobank,” Frontiers in Cardiovascular Medicine 8 (2021): 658726. [Internet]. [cited 2023 May 24], 10.3389/fcvm.2021.658726. 33889602 PMC8057521

[9] B. Coles , F. Zaccardi , S. Ling , M. J. Davies , N. J. Samani , and K. Khunti , “Cardiovascular Events and Mortality in People With and Without Type 2 Diabetes: An Observational Study in a Contemporary Multi‐Ethnic Population,” Journal of Diabetes Investigation 12, no. 7 (July 2021): 1175–1182, 10.1111/jdi.13464. 33206469 PMC8264396

[10] K. Panchal , C. Lawson , C. Chandramouli , C. Lam , K. Khunti , and F. Zaccardi , “Diabetes and Risk of Heart Failure in People With and Without Cardiovascular Disease: Systematic Review and Meta‐Analysis,” Diabetes Research and Clinical Practice 207 (December 15, 2023): 111054, 10.1016/j.diabres.2023.111054. 38104900

[11] T. A. McDonagh , M. Metra , M. Adamo , et al., “2021 ESC Guidelines for the Diagnosis and Treatment of Acute and Chronic Heart Failure,” European Heart Journal 42, no. 36 (September 21, 2021): 3599–3726, 10.1093/eurheartj/ehab368. 34447992

[12] E. Herrett , A. M. Gallagher , K. Bhaskaran , et al., “Data Resource Profile: Clinical Practice Research Datalink (CPRD),” International Journal of Epidemiology 44, no. 3 (June 2015): 827–836, 10.1093/ije/dyv098. 26050254 PMC4521131

[13] A. Wolf , D. Dedman , J. Campbell , et al., “Data Resource Profile: Clinical Practice Research Datalink (CPRD) Aurum,” International Journal of Epidemiology 48, no. 6 (December 1, 2019): 1740–1740g, 10.1093/ije/dyz034. 30859197 PMC6929522

[14] “The English Indices of Deprivation 2010,” https://www.gov.uk/government/uploads/system/uploads/attachment_data/file/6871/1871208.pdf.

[15] J. R. Brownrigg , V. Leo , J. Rose , et al., “Epidemiology of Cardiomyopathies and Incident Heart Failure in a Population‐Based Cohort Study,” Heart 108, no. 17 (September 2022): 1383–1391, 10.1136/heartjnl-2021-320181. 34969871

[16] K. Okoth , F. Crowe , T. Marshall , G. N. Thomas , K. Nirantharakumar , and N. J. Adderley , “Sex‐Specific Temporal Trends in the Incidence and Prevalence of Cardiovascular Disease in Young Adults: A Population‐Based Study Using UK Primary Care Data,” European Journal of Preventive Cardiology 29, no. 10 (August 5, 2022): 1387–1395, 10.1093/eurjpc/zwac024. 35139185

[17] S. I. Hemmo , A. Y. Naser , H. Alwafi , et al., “Hospital Admissions due to Ischemic Heart Diseases and Prescriptions of Cardiovascular Diseases Medications in England and Wales in the Past Two Decades,” International Journal of Environmental Research and Public Health 18, no. 13 (July 1, 2021): 7041, 10.3390/ijerph18137041. 34280978 PMC8297245

[18] E. Falaschetti , J. Mindell , C. Knott , and N. Poulter , “Hypertension Management in England: A Serial Cross‐Sectional Study From 1994 to 2011,” Lancet 383, no. 9932 (May 2014): 1912–1919, 10.1016/S0140-6736(14)60688-7. 24881995

[19] T. Jiao , R. W. Platt , A. Douros , and K. B. Filion , “Prescription Patterns for the Use of Antihypertensive Drugs for Primary Prevention Among Patients With Hypertension in the United Kingdom,” American Journal of Hypertension 35, no. 1 (January 5, 2022): 42–53, 10.1093/ajh/hpab137. 34448818 PMC8730500

[20] S. L. Lay‐Flurrie , J. P. Sheppard , R. J. Stevens , et al., “Impact of Changes to National Hypertension Guidelines on Hypertension Management and Outcomes in the United Kingdom,” Hypertension 75, no. 2 (February 2020): 356–364, 10.1161/HYPERTENSIONAHA.119.13926. 31865798 PMC7055938

[21] B. Zhou , G. Danaei , G. A. Stevens , et al., “Long‐Term and Recent Trends in Hypertension Awareness, Treatment, and Control in 12 High‐Income Countries: An Analysis of 123 Nationally Representative Surveys,” Lancet 394, no. 10199 (August 2019): 639–651, 10.1016/S0140-6736(19)31145-6. 31327564 PMC6717084

[22] B. M. Opazo , D. Gillespie , R. Pryce , et al., “Understanding Long‐Term Trends in Smoking in England, 1972–2019: An Age–Period–Cohort Approach,” Addiction 117, no. 5 (May 2022): 1392–1403, 10.1111/add.15696. 34590368

[23] H. F. Chen , Y. H. Chang , H. J. Lo , et al., “Incidence of Idiopathic Cardiomyopathy in Patients With Type 2 Diabetes in Taiwan: Age, Sex, and Urbanization Status‐Stratified Analysis,” Cardiovascular Diabetology 19, no. 1 (December 2020): 177, 10.1186/s12933-020-01144-y. 33054769 PMC7558694

[24] G. Querio , S. Antoniotti , F. Geddo , et al., “Ischemic Heart Disease and Cardioprotection: Focus on Estrogenic Hormonal Setting and Microvascular Health,” Vascular Pharmacology 141 (December 2021): 106921, 10.1016/j.vph.2021.106921. 34592428

[25] D. Xiang , Y. Liu , S. Zhou , E. Zhou , and Y. Wang , “Protective Effects of Estrogen on Cardiovascular Disease Mediated by Oxidative Stress,” S. D'Adamo, ed.,” Oxidative Medicine and Cellular Longevity 2021 (June 28, 2021): 1–15, 10.1155/2021/5523516. PMC8260319 34257804

[26] A. Iorga , C. M. Cunningham , S. Moazeni , G. Ruffenach , S. Umar , and M. Eghbali , “The Protective Role of Estrogen and Estrogen Receptors in Cardiovascular Disease and the Controversial Use of Estrogen Therapy,” Biology of Sex Differences 8, no. 1 (December 2017): 33, 10.1186/s13293-017-0152-8. 29065927 PMC5655818

[27] S. S. Zghebi , D. T. Steinke , M. K. Rutter , and D. M. Ashcroft , “Eleven‐Year Multimorbidity Burden Among 637 255 People With and Without Type 2 Diabetes: A Population‐Based Study Using Primary Care and Linked Hospitalisation Data,” BMJ Open 10, no. 7 (July 2020): e033866, 10.1136/bmjopen-2019-033866. PMC7358107 32611677

[28] H. Wada , K. Miyauchi , and H. Daida , “Gender Differences in the Clinical Features and Outcomes of Patients With Coronary Artery Disease,” Expert Review of Cardiovascular Therapy 17, no. 2 (February 2019): 127–133, 10.1080/14779072.2019.1561277. 30569774

[29] A. Nordström , J. Hadrévi , T. Olsson , P. W. Franks , and P. Nordström , “Higher Prevalence of Type 2 Diabetes in Men Than in Women Is Associated With Differences in Visceral Fat Mass,” Journal of Clinical Endocrinology and Metabolism 101, no. 10 (October 2016): 3740–3746, 10.1210/jc.2016-1915. 27490920

[30] K. Janochova , M. Haluzik , and M. Buzga , “Visceral Fat and Insulin Resistance ‐ What We Know?,” Biomedical Papers 163, no. 1 (February 18, 2019): 19–27, 10.5507/bp.2018.062. 30398218

[31] S. G. Abdissa , W. Deressa , and A. J. Shah , “Incidence of Heart Failure Among Diabetic Patients With Ischemic Heart Disease: A Cohort Study,” BMC Cardiovascular Disorders 20, no. 1 (December 2020): 181, 10.1186/s12872-020-01457-6. 32306907 PMC7169007

[32] S. De Lusignan , S. T. Liaw , D. Dedman , K. Khunti , K. Sadek , and S. Jones , “An Algorithm to Improve Diagnostic Accuracy in Diabetes in Computerised Problem Orientated Medical Records (POMR) Compared With an Established Algorithm Developed in Episode Orientated Records (EOMR),” Journal of Innovation in Health Informatics 22, no. 2 (June 5, 2015): 255–264, 10.14236/jhi.v22i2.79. 26245239

[33] R. Persson , C. Vasilakis‐Scaramozza , K. W. Hagberg , et al., “CPRD Aurum Database: Assessment of Data Quality and Completeness of Three Important Comorbidities,” Pharmacoepidemiology and Drug Safety 29, no. 11 (November 2020): 1456–1464, 10.1002/pds.5135. 32986901

