# Supplementary material for: Risk of Ischaemic and Non‐Ischaemic Heart Failure in People With Type 2 Diabetes: Observational Study in 1.6 Million People in England
Source: Diabetes Metab Res Rev. 2025 Jul 28;41(6):e70072. doi: 10.1002/dmrr.70072 (PMC12304495; doi:10.1002/dmrr.70072)
Supplement: Supplementary file 1 — Supporting Information S1 [file DMRR-41-e70072-s001.docx]

**SUPPLEMENTARY MATERIAL**

**Risk of ischaemic and non-ischaemic heart failure in people with type 2 diabetes: observational study in 1.6 million people in England**

**Contents**

[**Table S1: Matching details** 2](#_Toc197373044)

[**Table S2: Missing data at baseline** 3](#_Toc197373045)

[**Table S3: Baseline characteristics by type of incident heart failure in women** 4](#_Toc197373046)

[**Table S4: Baseline characteristics by type of incident heart failure in men** 5](#_Toc197373047)

[**Table S5: Sex-stratified rates of incident heart failure – heart failure defined as heart failure hospitalisation or heart failure death** 6](#_Toc197373048)

[**Table S6: Sex-stratified hazard ratios of incident heart failure – heart failure defined as heart failure hospitalisation or heart failure death** 7](#_Toc197373049)

[**Table S7: Sex-stratified rates of incident heart failure – including people with prevalent ischaemic heart disease at baseline** 8](#_Toc197373050)

[**Table S8: Sex-stratified hazard ratios of incident heart failure – including people with prevalent ischaemic heart disease at baseline** 9](#_Toc197373051)

[**Figure S1: Flowchart of cohort definition in CPRD GOLD** 10](#_Toc197373052)

[**Figure S2: Flowchart of cohort definition in CPRD Aurum** 11](#_Toc197373053)

[**RECORD CHECKLIST** 12](#_Toc197373054)

# **Table S1: Matching details**

| **Initial Cohort** | **Unmatched** | **Matched 1:1 ratio** | **Matched 1:2 ratio** | **Matched 1:3 ratio** | **Matched 1:4 ratio** |
| --- | --- | --- | --- | --- | --- |
| With type 2 diabetes  Without diabetes | 17,113  0 | 4,211 4,211 | 4,095  8,190 | 4,341  13,023 | 916,851  3,667,404 |

The matching process was implemented (Figures S1 and S2):

- before excluding people aged <18 years old

- before excluding those if date of death, HES last linkage date, CPRD last collection date, CPRD transfer out date, or end date was at or prior to index date

- prior to excluding individuals with sex no male or no female

- before excluding individuals with selected conditions at or prior to the index date (i.e., prevalent condition: heart failure, ischaemic heart disease, stroke and peripheral vascular disease)

- prior to excluding those prescribed glucose-lowering drugs and without diabetes

- prior to excluding those with missing data on ethnicity, index of multiple deprivation, systolic blood pressure, smoking and body mass index.

Abbreviations: SD = standard deviation. Further details on the final cohort definition are shown in Figures S1 and S2.

# **Table S2: Missing data at baseline**

|  | **Total**  **(n = 1,621,090)** | | **People with type 2 diabetes**  **(n = 532,185)** | | **People without diabetes**  **(n = 1,088,905)** | |
| --- | --- | --- | --- | --- | --- | --- |
|  | **Missing (n)** | **Missing (%)** | **Missing (n)** | **Missing (%)** | **Missing (n)** | **Missing (%)** |
| Total cholesterol | 493,457 | 30.44 | 82,596 | 15.52 | 410,861 | 37.73 |
| Alcohol intake status | 232,423 | 14.34 | 68,162 | 12.81 | 164,261 | 15.08 |

Abbreviations: n = number of people.

Missing data for total cholesterol and alcohol intake status in the cohort of people without missing data on ethnicity, IMD, systolic BP, smoking status, BMI (Figure S1 and S2).

# **Table S3: Baseline characteristics by type of incident heart failure in women**

|  | **Ischaemic Heart Failure** | | **Non-ischaemic Heart Failure** | |
| --- | --- | --- | --- | --- |
|  | **Women with**  **type 2 diabetes  n = 1,967** | **Women**  **without diabetes n = 2,774** | **Women with**  **type 2 diabetes  n = 7,529** | **Women**  **without diabetes n = 11,812** |
| Age (years) | 68.22 (11.49) | 71.44 (8.93) | 70.96 (11.48) | 73.19 (8.89) |
| Ethnicity   White  South Asian   Black  Mixed/Other   Unknown | 1,706 (86.73)  123 (6.25)  57 (2.90)  60 (3.05)  21 (1.07) | 2,613 (94.20)  49 (1.77)  29 (1.05)  27 (0.97)  56 (2.02) | 6,650 (88.33)  271 (3.60)  270 (3.59)  141 (1.87)  197 (2.62) | 10,969 (92.86)  171 (1.45)  194 (1.64)  142 (1.20)  336 (2.84) |
| IMD quintile  1 (least deprived)  2  3   4  5 (most deprived) | 342 (17.39)  351 (17.84) 373 (18.96)  401 (20.39)  500 (25.42) | 512 (18.46)  553 (19.94)  537 (19.36)  604 (21.77)  568 (20.48) | 1,224 (16.26)  1,366 (18.14) 1,509 (20.04)  1,618 (21.49)  1,812 (24.07) | 2,267 (19.19)  2,436 (20.62)  2,396 (20.28)  2,364 (20.01)  2,349 (19.89) |
| Smoking status   Ever-smoker   Non-smoker | 923 (46.92)  1,044 (53.08) | 1,288 (46.43)  1,486 (53.57) | 3,334 (44.28)  4,195 (55.72) | 4,975 (42.12)  6,837 (57.88) |
| Alcohol intake status  Current  Ex-drinker   Never | 1,433 (82.45)  $  297 (17.09) | 2,085 (85.00) $ 361 (14.72) | 5,663 (84.00)  36 (0.53)  1,043 (15.47) | 8,910 (84.82)  55 (0.52)  1,540 (14.66) |
| BMI (kg/m^2^) | 31.86 ± 6.84 | 27.16 ± 5.72 | 32.93 ± 7.91 | 27.91± 6.22 |
| Systolic BP (mmHg) | 145.39 ± 18.97 | 143.66 ± 18.63 | 144.01 ± 19.40 | 141.64 ± 17.80 |
| Total cholesterol (mmol/l) | 4.92 ± 1.67 | 5.05 ± 1.58 | 4.71 ± 1.58 | 4.81 ± 1.52 |
| **Comorbidities** | | | | |
| Anaemia | 152 (7.73) | 206 (7.43) | 725 (9.63) | 1,048 (8.87) |
| Asthma | 416 (21.15) | 415 (14.96) | 1,509 (20.04) | 1,842 (15.59) |
| Atrial fibrillation | 152 (7.73) | 166 (5.98) | 1,064 (14.13) | 1,136 (9.62) |
| Cancer | 269 (13.68) | 414 (14.92) | 1,277 (16.96) | 1,982 (16.78) |
| Chronic kidney disease | 462 (23.49) | 608 (21.92) | 2,049 (27.21) | 2,850 (24.13) |
| Chronic liver disease | 33 (1.68) | 17 (0.61) | 118 (1.57) | 111 (0.94) |
| COPD | 132 (6.71) | 188 (6.78) | 706 (9.38) | 898 (7.60) |
| Dementia | $ | 22 (0.79) | 95 (1.26) | 132 (1.12) |
| Depression | 418 (21.25) | 517 (18.64) | 1,561 (20.73) | 2,128 (18.02) |
| Hypertension | 1,344 (68.33) | 1,436 (51.77) | 5,348 (71.03) | 6,454 (54.64) |
| Osteoarthritis | 512 (26.03) | 588 (21.20) | 2,133 (28.33) | 2,823 (23.90) |
| Rheumatoid arthritis | 56 (2.85) | 102 (3.68) | 258 (3.43) | 410 (3.47) |
| Thyroid disorders | 291 (14.79) | 347 (12.51) | 1,138 (15.11) | 1,528 (12.94) |
| **Medication prescription** | | | | |
| Antihypertensive drugs | 1,589 (80.78) | 1,840 (66.33) | 6,413 (85.18) | 8,359 (70.77) |
| Antiplatelet drugs | 576 (29.28) | 667 (24.04) | 2,281 (30.30) | 2,955 (25.02) |
| Digoxin | 101 (5.13) | 101 (3.64) | 651 (8.65) | 570 (4.83) |
| Lipid lowering drugs | 656 (33.35) | 583 (21.02) | 2,567 (34.09) | 2,470 (20.91) |

Abbreviations: BMI = body mass index, COPD = chronic obstructive pulmonary disease, BP = blood pressure, IMD = index of multiple deprivation.

All categorical variables are reported as number (proportion, %) and continuous variables as mean (standard deviation).

Cohort of people without missing data on ethnicity, IMD, systolic BP, smoking status, BMI (Figure S1 and S2).

^$^ Suppressed (≤10).

# **Table S4: Baseline characteristics by type of incident heart failure in men**

|  | **Ischaemic Heart Failure** | | **Non-ischaemic Heart Failure** | |
| --- | --- | --- | --- | --- |
|  | **Men with**  **type 2 diabetes  n = 3,079** | **Men  without diabetes n = 4,227** | **Men with**  **type 2 diabetes  n = 7,441** | **Men  without diabetes n = 11,022** |
| Age (years) | 65.04 (10.94) | 67.56 (9.28) | 67.86 (11.65) | 69.81 (9.47) |
| Ethnicity   White  South Asian   Black  Mixed/Other   Unknown | 2,703 (87.79)  177 (5.75) 57 (1.85)  92 (2.99)  50 (1.62) | 3,948 (93.40)  88 (2.08)  49 (1.16)  65 (1.54)  77 (1.82) | 6.625 (89.03)  176 (2.37)  272 (3.66)  164 (2.20)  204 (2.74) | 10,235 (92.86)  119 (1.08)  182 (1.65)  129 (1.17)  357 (3.24) |
| IMD quintile  1 (least deprived)  2  3   4  5 (most deprived) | 529 (17.18)  600 (19.49)  597 (19.39)  646 (20.98)  707 (22.96) | 897 (21.22)  934 (22.10)  803 (19.00)  825 (19.52)  768 (18.17) | 1,355 (18.21)  1,445 (19.42)  1,465 (19.69)  1,521 (20.44)  1,655 (22.24) | 2,358 (21.39)  2,437 (22.11)  2,172 (19.71)  2,113 (19.17)  1,942 (17.62) |
| Smoking status   Ever-smoker   Non-smoker | 1,925 (62.52)  1,154 (37.48) | 2,443 (57.80)  1,784 (42.20) | 4,549 (61.13) 2,892 (38.87) | 6,329 (57.42)  4,693 (42.58) |
| Alcohol intake status  Current  Ex-drinker   Never | 2,438 (91.04)  25 (0.93)  215 (8.03) | 3,382 (92.08)  20 (0.54)  271 (7.38) | 6,122 (92.05)  59 (0.89)  470 (7.07) | 9,018 (92.56)  63 (0.65)  662 (6.79) |
| BMI (kg/m^2^) | 30.83 ± 5.65 | 27.14 ± 4.21 | 31.93 ± 6.59 | 27.47 ± 4.77 |
| Systolic BP (mmHg) | 144.81 ± 18.80 | 140.56 ± 17.03 | 143.55 ± 18.51 | 140.11 ± 17.31 |
| Total cholesterol (mmol/l) | 4.83 ± 1.67 | 4.76 ± 1.33 | 4.50 ± 1.49 | 4.53 ± 1.31 |
| **Comorbidities** | | | | |
| Anaemia | 94 (3.05) | 151 (3.57) | 384 (5.16) | 540 (4.90) |
| Asthma | 425 (13.80) | 500 (11.83) | 1,066 (14.33) | 1,536 (13.94) |
| Atrial fibrillation | 181 (5.88) | 244 (5.77) | 1,046 (14.06) | 1,266 (11.49) |
| Cancer | 311 (10.10) | 524 (12.40) | 1,043 (14.02) | 1,671 (15.16) |
| Chronic kidney disease | 412 (13.38) | 518 (12.25) | 1,278 (17.18) | 1,530 (13.88) |
| Chronic liver disease | 28 (0.91) | 27 (0.64) | 152 (2.04) | 130 (1.18) |
| COPD | 221 (7.18) | 282 (6.67) | 803 (10.79) | 1,100 (9.98) |
| Dementia | 17 (0.55) | 16 (0.38) | 44 (0.59) | 90 (0.82) |
| Depression | 383 (12.44) | 441 (10.43) | 853 (11.46) | 1,162 (10.54) |
| Hypertension | 1,737 (56.41) | 1,751 (41.42) | 4,740 (63.70) | 5,021 (45.55) |
| Osteoarthritis | 510 (16.56) | 629 (14.88) | 1,437 (19.31) | 1,788 (16.22) |
| Rheumatoid arthritis | 45 (1.46) | 61 (1.44) | 132 (1.77) | 203 (1.84) |
| Thyroid disorders | 120 (3.90) | 111 (2.63) | 279 (3.75) | 334 (3.03) |
| **Medication prescription** | | | | |
| Antihypertensive drugs | 2,086 (67.75) | 2,319 (54.86) | 5,608 (75.37) | 6,572 (59.63) |
| Antiplatelet drugs | 740 (24.03) | 882 (20.87) | 2,224 (29.89) | 2,555 (23.18) |
| Digoxin | 92 (2.99) | 100 (2.37) | 537 (7.22) | 514 (4.66) |
| Lipid lowering drugs | 978 (31.76) | 804 (19.02) | 2,600 (34.94) | 2,264 (20.54) |

Abbreviations: BMI = body mass index, COPD = chronic obstructive pulmonary disease, BP = blood pressure, IMD = index of multiple deprivation.

All categorical variables are reported as number (proportion, %) and continuous variables as mean (standard deviation).

Cohort of people without missing data on ethnicity, IMD, systolic BP, smoking status, BMI (Figure S1 and S2).

# **Table S5: Sex-stratified rates of incident heart failure – heart failure defined as heart failure hospitalisation or heart failure death**

| **Outcome, sex, group** | **Events/N** | **Crude IR per 1,000 person-years (95% CI)** | **Age-standardised IR per 1,000 person-years (95% CI)** | **Crude IRR  (95% CI)** | **Age-adjusted IRR**  **(95% CI)** |
| --- | --- | --- | --- | --- | --- |
| **Heart Failure** | | | | | |
| *Women*  With type 2 diabetes   Without diabetes | 6,730/274,762 11,182/847,393 | 3.27 (3.19-3.34)  1.69 (1.66-1.72) | 1.86 (1.80-1.92)  0.99 (0.97-1.02) | 1.94 (1.88-1.99)  REF | 1.88 (1.82-1.93)  REF |
| *Men*  With type 2 diabetes   Without diabetes | 6,617/257,458 10,524 /721,852 | 3.30 (3.22-3.38)  1.82 (1.78-1.85) | 2.51 (2.43-2.58)  1.36 (1.33-1.39) | 1.82 (1.76-1.88)  REF | 1.84 (1.78-1.90)  REF |
| **Ischaemic Heart Failure** | | | | | |
| *Women*  With type 2 diabetes   Without diabetes | 1,126/274,762  1,532/847,393 | 0.54 (0.51-0.57) 0.23 (0.22-0.24) | 0.38 (0.35-0.41)  0.16 (0.15-0.17) | 2.36 (2.18-2.54)  REF | 2.32 (2.15-2.51) REF |
| *Men*  With type 2 diabetes   Without diabetes | 1,625/257,458 2,316/721,852 | 0.81 (0.77-0.85)  0.40 (0.38-0.41) | 0.69 (0.65-0.72)  0.33 (0.32-0.35) | 2.02 (1.90-2.16)  REF | 2.06 (1.94-2.20)  REF |
| **Non-ischaemic Heart Failure** | | | | | |
| *Women*  With type 2 diabetes   Without diabetes | 5,604/274,762  9,650/847,393 | 2.72 (2.64-2.79)  1.46 (1.43-1.49) | 1.48 (1.43-1.53)  0.82 (0.80-0.84) | 1.87 (1.81-1.93)  REF | 1.80 (1.74-1.86)  REF |
| *Men*  With type 2 diabetes   Without diabetes | 4,992/257,458  8,208/721,852 | 2.49 (2.42-2.55)  1.41 (1.38-1.45) | 1.81 (1.75-1.87)  1.02 (1.00-1.05) | 1.76 (1.70-1.82) REF | 1.77 (1.71-1.83)  REF |

Abbreviations: N = number of people, IR = incidence rate, IRR = incidence rate ratio, CI = confidence interval, REF = reference group.

Age-standardised rates were estimated at mean age of the total population at 60 years old.

# **Table S6: Sex-stratified hazard ratios of incident heart failure – heart failure defined as heart failure hospitalisation or heart failure death**

| **Outcome, sex** | **Model 1** | | **Model 2** | | **Model 3** | | **Model 4** | | **Model 5** | |
| --- | --- | --- | --- | --- | --- | --- | --- | --- | --- | --- |
|  | **Events/N** | **HR (95% CI)** | **Events/N** | **HR (95% CI)** | **Events/N** | **HR (95% CI)** | **Events/N** | **HR (95% CI)** | **Events/N** | **HR (95% CI)** |
| **Heart Failure** | | | | | | | | | | |
| Women  Men | 17,912/1,122,155  17,141/974,310 | 1.98 (1.92-2.05)  1.85 (1.80-1.91) | 17,912/1,122,155  17,141/974,310 | 1.98 (1.92-2.04)  1.91 (1.85-1.97) | 15,928/955,420 15,143/830,737 | 1.88 (1.82-1.95)  1.84 (1.78-1.90) | 10,218/585,178 9,718/541,458 | 1.25 (1.20-1.31)  1.33 (1.27-1.39) | 10,218/585,178 9,718/541,458 | 1.23 (1.18-1.29)  1.33 (1.27-1.39) |
| **Ischaemic Heart Failure** | | | | | | | | | | |
| Women  Men | 2,658/1,122,155 3,941/979,310 | 2.45 (2.27-2.64)  2.08 (1.95-2.22) | 2,658/1,122,155 3,941/979,310 | 2.50 (2.31-2.70) 2.18 (2.04-2.32) | 2,381/955,420 3,487/830,737 | 2.34 (2.16-2.54)  2.07 (1.93-2.21) | 1,517/585,178  2,230/541,458 | 1.68 (1.50-1.87)  1.59 (1.46-1.74) | 1,517/585,178  2,230/541,458 | 1.63 (1.47-1.82) 1.58 (1.44-1.73) |
| **Non-ischaemic Heart Failure** | | | | | | | | | | |
| Women  Men | 15,254/1,122,155  13,200/979,310 | 1.91 (1.85-1.97)  1.78 (1.72-1.85) | 15,254/1,122,155  13,200/979,310 | 1.89 (1.83-1.95)  1.83 (1.77-1.89) | 13,547/955,420 11,656/830,737 | 1.81 (1.74-1.87)  1.76 (1.70-1.83) | 8,701/585,178 7,488/541,458 | 1.19 (1.14-1.24)  1.26 (1.20-1.32) | 8,701/585,178 7,488/541,458 | 1.17 (1.12-1.23)  1.26 (1.20-1.32) |

Reference group for total = people without diabetes, women = women without diabetes, men = men without diabetes.

Model 1) Unadjusted.

Model 2) Adjusted for age.

Model 3) Adjusted for age, index of multiple deprivation, ethnicity, smoking, and alcohol intake status.
Model 4) Adjusted for age, index of multiple deprivation, ethnicity, smoking, alcohol intake status and comorbidities (anaemia, asthma, atrial fibrillation, cancer, chronic kidney disease,

chronic liver disease, chronic obstructive pulmonary disease, dementia, depression, hypertension, osteoarthritis, rheumatoid arthritis, thyroid disorders), body mass index, systolic blood pressure, total cholesterol level.
Model 5) Adjusted for age, index of multiple deprivation, ethnicity, smoker status, alcohol intake status, comorbidities (anaemia, asthma, atrial fibrillation, cancer, chronic kidney disease, chronic liver disease, chronic obstructive pulmonary disease, dementia, depression, hypertension, osteoarthritis, rheumatoid arthritis, thyroid disorders), body mass index, systolic blood pressure, total cholesterol level and prescriptions (antihypertensive medications, antiplatelets medications, digoxin, and lipid-lowering medications).

Abbreviations: HR = hazard ratio, CI = confidence intervals, HF = heart failure, N = number of people.

# **Table S7: Sex-stratified rates of incident heart failure – including people with prevalent ischaemic heart disease at baseline**

| **Outcome, sex, group** | **Events/N** | **Crude IR per 1,000 person-years (95% CI)** | **Age-standardised IR per 1,000 person-years (95% CI)** | **Crude IRR  (95% CI)** | **Age-adjusted IRR**  **(95% CI)** |
| --- | --- | --- | --- | --- | --- |
| **Heart Failure** | | | | | |
| *Women*  With type 2 diabetes   Without diabetes | 13,108/312,580  18,257/629,588 | 6.65 (6.53-6.76)  4.14 (4.08-4.20) | 3.71 (3.62-3.79)  2.26 (2.22-2.30) | 1.60 (1.57-1.64)  REF | 1.64 (1.60-1.68)  REF |
| *Men*   With type 2 diabetes   Without diabetes | 17,006/318,789 21,804/581,732 | 8.25 (8.12-8.37)  5.36 (5.28-5.43) | 5.83 (5.73-5.94)  3.64 (3.59-3.70) | 1.54 (1.51-1.57)  REF | 1.60 (1.57-1.63)  REF |
| **Ischaemic Heart Failure** | | | | | |
| *Women*   With type 2 diabetes   Without diabetes | 5,579/312,580  6,445/629,588 | 2.80 (2.73-2.87)  1.45 (1.42-1.49) | 1.50 (1.45-1.56)  0.77 (0.74-0.79) | 1.93 (1.86-2.00)  REF | 1.96 (1.89-2.04)  REF |
| *Men*  With type 2 diabetes   Without diabetes | 9,565/318,789 10,782581,732 | 4.59 (4.50-4.68)  2.63 (2.58-2.68) | 3.21 (3.13-3.29) 1.77 (1.73-1.80) | 1.75 (1.70-1.80)  REF | 1.82 (1.77-1.87)  REF |
| **Non-ischaemic Heart Failure** | | | | | |
| *Women*  With type 2 diabetes   Without diabetes | 7,529/312,580 11,812/629,588 | 3.79 (3.70-3.87)  2.67 (2.62-2.72) | 2.16 (2.09-2.22)  1.49 (1.46-1.52) | 1.42 (1.38-1.46)  REF | 1.45 (1.41-1.49)  REF |
| *Men*   With type 2 diabetes   Without diabetes | 7,441/318,789  11,022/581,732 | 3.56 (3.48-3.64)  2.68 (2.63-2.73) | 2.55 (2.48-2.62)  1.85 (1.81-1.89) | 1.32 (1.29-1.36)  REF | 1.38 (1.34-1.42)  REF |

Abbreviations: N = number of people, IR = incidence rate, IRR = incidence rate ratio, CI = confidence interval, REF = reference group.

Age-standardised rates were estimated at mean age of the total population at 60 years old.

# **Table S8: Sex-stratified hazard ratios of incident heart failure – including people with prevalent ischaemic heart disease at baseline**

| **Outcome, sex** | **Model 1** | | **Model 2** | | **Model 3** | | **Model 4** | | **Model 5** | |
| --- | --- | --- | --- | --- | --- | --- | --- | --- | --- | --- |
|  | **Events/N** | **HR (95% CI)** | **Events/N** | **HR (95% CI)** | **Events/N** | **HR (95% CI)** | **Events/N** | **HR (95% CI)** | **Events/N** | **HR (95% CI)** |
| **Heart Failure** | | | | | | | | | | |
| Women  Men | 31,365/942,168  38,810/900,521 | 1.66 (1.63-1.70)  1.58 (1.55-1.61) | 31,365/942,168  38,810/900,521 | 1.73 (1.69-1.77)  1.67 (1.64-1.70) | 28,032/813,197  34,429/769,710 | 1.67 (1.63-1.71)  1.63 (1.59-1.66) | 20,474/587,278 26,528/588,500 | 1.18 (1.15-1.22) 1.22 (1.19-1.25) | 20,474/587,278 26,528/588,500 | 1.15 (1.12-1.18) 1.19 (1.16-1.22) |
| **Ischaemic Heart Failure** | | | | | | | | | | |
| Women Men | 12,024/942,168 20,347/900,521 | 1.99 (1.92-2.06)  1.79 (1.74-1.84) | 12,024/942,168 20,347/900,521 | 2.06 (1.99-2.14)  1.89 (1.84-1.94) | 10,785/813,197 18,035/769,710 | 1.99 (1.92-2.07)  1.83 (1.78-1.88) | 8,550/587,278 14,926/588,500 | 1.35 (1.29-1.41)  1.35 (1.31-1.40) | 8,550/587,278 14,926/588,500 | 1.24 (1.18-1.30)  1.26 (1.22-1.31) |
| **Non-ischaemic Heart Failure** | | | | | | | | | | |
| Women   Men | 19,341/942,168 18,463/900,521 | 1.47 (1.43-1.51)  1.36 (1.32-1.40) | 19,341/942,168 18,463/900,521 | 1.53 (1.48-1.57)  1.43 (1.39-1.47) | 17,247/813,197 16,394/769,710 | 1.48 (1.43-1.52)  1.40 (1.36-1.45) | 11,924/587,278 11,602/588,500 | 1.07 (1.03-1.11)  1.06 (1.02-1.10) | 11,924/587,278 11,602/588,500 | 1.09 (1.05-1.14)  1.10 (1.06-1.15) |

Model 1) Unadjusted.

Model 2) Adjusted for age.

Model 3) Adjusted for age, index of multiple deprivation, ethnicity, smoking, and alcohol intake status.
Model 4) Adjusted for age, index of multiple deprivation, ethnicity, smoking, alcohol intake status and comorbidities (anaemia, asthma, atrial fibrillation, cancer, chronic kidney disease, chronic liver disease, chronic obstructive pulmonary disease, dementia, depression, hypertension, osteoarthritis, rheumatoid arthritis, thyroid disorders), body mass index, systolic blood pressure, total cholesterol level.
Model 5) Adjusted for age, index of multiple deprivation, ethnicity, smoker status, alcohol intake status, comorbidities (anaemia, asthma, atrial fibrillation, cancer, chronic kidney disease, chronic liver disease, chronic obstructive pulmonary disease, dementia, depression, hypertension, osteoarthritis, rheumatoid arthritis, thyroid disorders), body mass index, systolic blood pressure, total cholesterol level and prescriptions (antihypertensive medications, antiplatelets medications, digoxin, and lipid-lowering medications).

Abbreviations: HR = hazard ratio, CI = confidence intervals, N = number of people.

# **Figure S1: Flowchart of cohort definition in CPRD GOLD**


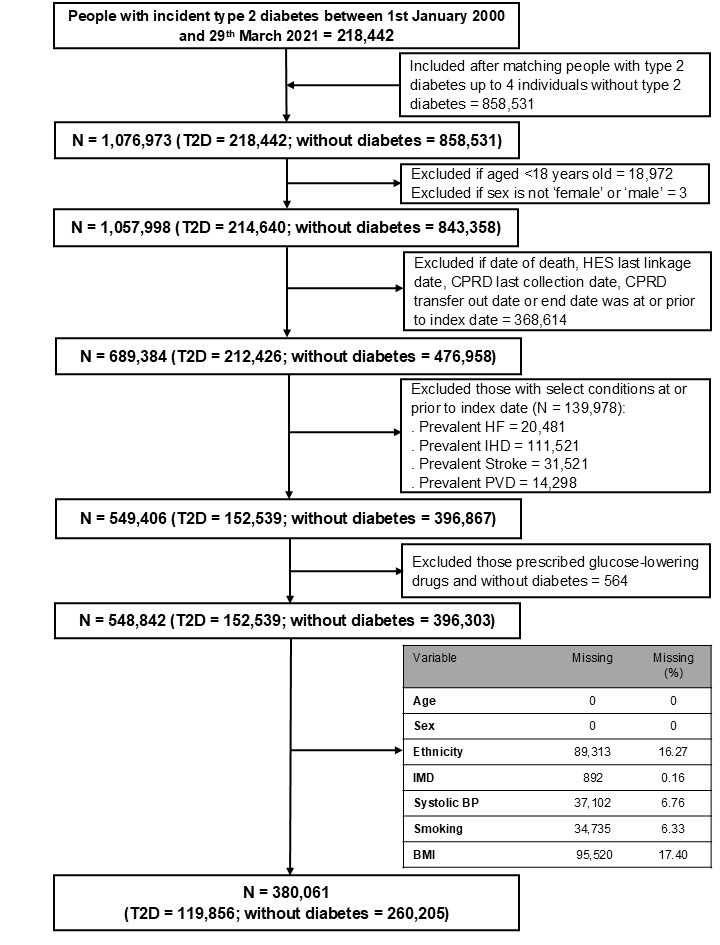
Abbreviations: T2D = type 2 diabetes, CPRD = Clinical Research Practice datalink data, CVD = cardiovascular disease, N = total participants, IMD = index of multiple deprivation, MI = myocardial infarction, IHD = ischaemic heart disease, PVD = peripheral vascular disease, BMI = body mass index, BP = blood pressure. Details of the matching process are reported in Table S2.

#
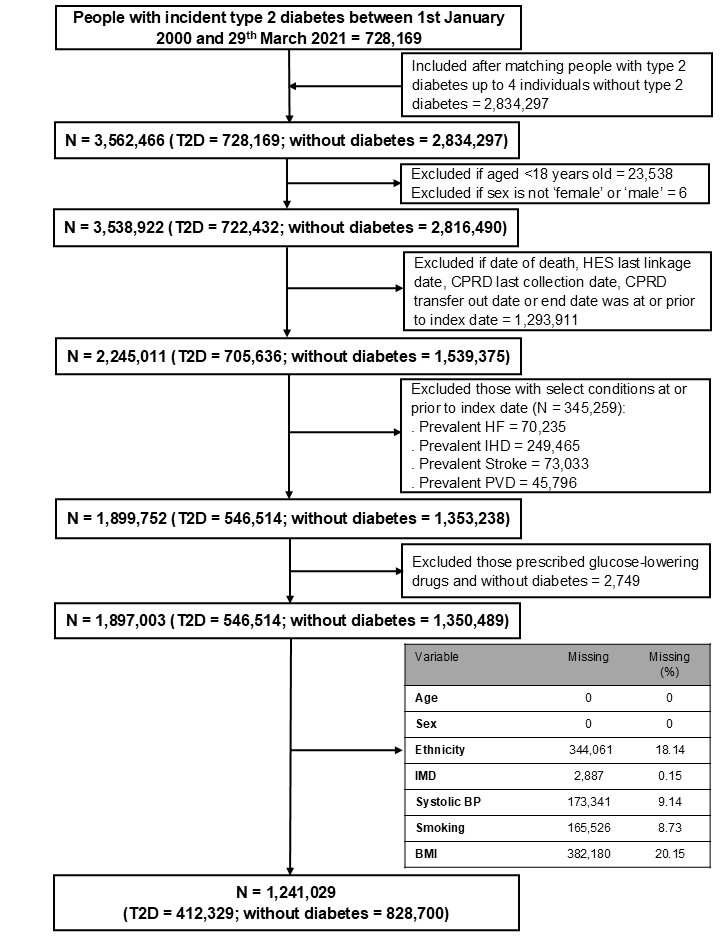
**Figure S2: Flowchart of cohort definition in CPRD Aurum**

Abbreviations: T2D = type 2 diabetes, CPRD = Clinical Research Practice datalink data, CVD = cardiovascular disease, N = total participants, IMD = index of multiple deprivation, MI = myocardial infarction, IHD = ischaemic heart disease, PVD = peripheral vascular disease, BMI = body mass index, BP = blood pressure. Details of the matching process are reported in Table S2.

# **RECORD CHECKLIST**

|  | **Item No.** | **STROBE items** | **Location in manuscript where items are reported** | **RECORD items** | **Location in manuscript where items are reported** |
| --- | --- | --- | --- | --- | --- |
| **Title and abstract** | | | | | |
|  | 1 | (a) Indicate the study’s design with a commonly used term in the title or the abstract (b) Provide in the abstract an informative and balanced summary of what was done and what was found | Page 1-4 | RECORD 1.1: The type of data used should be specified in the title or abstract. When possible, the name of the databases used should be included.  RECORD 1.2: If applicable, the geographic region and timeframe within which the study took place should be reported in the title or abstract.  RECORD 1.3: If linkage between databases was conducted for the study, this should be clearly stated in the title or abstract. | Page 1-3  Page 1-2  Page 1-2 |
| **Introduction** | | | | | |
| Background rationale | 2 | Explain the scientific background and rationale for the investigation being reported | Page 5 |  |  |
| Objectives | 3 | State specific objectives, including any prespecified hypotheses | Page 5 |  |  |
| **Methods** | | | | | |
| Study Design | 4 | Present key elements of study design early in the paper | P Page 6 |  |  |
| Setting | 5 | Describe the setting, locations, and relevant dates, including periods of recruitment, exposure, follow-up, and data collection | Page 6-7 |  |  |
| Participants | 6 | *(a) Cohort study* - Give the eligibility criteria, and the sources and methods of selection of participants. Describe methods of follow-up  *Case-control study* - Give the eligibility criteria, and the sources and methods of case ascertainment and control selection. Give the rationale for the choice of cases and controls  *Cross-sectional study* - Give the eligibility criteria, and the sources and methods of selection of participants  *(b) Cohort study* - For matched studies, give matching criteria and number of exposed and unexposed  *Case-control study* - For matched studies, give matching criteria and the number of controls per case | Page 6-8  Page 6 | RECORD 6.1: The methods of study population selection (such as codes or algorithms used to identify subjects) should be listed in detail. If this is not possible, an explanation should be provided.  RECORD 6.2: Any validation studies of the codes or algorithms used to select the population should be referenced. If validation was conducted for this study and not published elsewhere, detailed methods and results should be provided.  RECORD 6.3: If the study involved linkage of databases, consider use of a flow diagram or other graphical display to demonstrate the data linkage process, including the number of individuals with linked data at each stage. | Page 6-8;  Figure S1; Figure S2.  Page 6  Page 6-8 |
| Variables | 7 | Clearly define all outcomes, exposures, predictors, potential confounders, and effect modifiers. Give diagnostic criteria, if applicable. | Page 6-7 | RECORD 7.1: A complete list of codes and algorithms used to classify exposures, outcomes, confounders, and effect modifiers should be provided. If these cannot be reported, an explanation should be provided. | Page 6 |
| Data sources/ measurement | 8 | For each variable of interest, give sources of data and details of methods of assessment (measurement).  Describe comparability of assessment methods if there is more than one group | Page 6-8 |  |  |
| Bias | 9 | Describe any efforts to address potential sources of bias | Page 6-8 |  |  |
| Study size | 10 | Explain how the study size was arrived at | Page 6-7;  Figure S1; Figure S2. |  |  |
| Quantitative variables | 11 | Explain how quantitative variables were handled in the analyses. If applicable, describe which groupings were chosen, and why | Page 6-8 |  |  |
| Statistical methods | 12 | (a) Describe all statistical methods, including those used to control for confounding  (b) Describe any methods used to examine subgroups and interactions  (c) Explain how missing data were addressed  (d) *Cohort study* - If applicable, explain how loss to follow-up was addressed  (e) Describe any sensitivity analyses | Page 6-9    Page 7-9  Page 7-8  Page 6-8  Page 9 |  |  |
| Data access and cleaning methods |  | .. |  | RECORD 12.1: Authors should describe the extent to which the investigators had access to the database population used to create the study population.  RECORD 12.2: Authors should provide information on the data cleaning methods used in the study. | Page 6-9  Page 6-9 |
| Linkage |  | .. |  | RECORD 12.3: State whether the study included person-level, institutional-level, or other data linkage across two or more databases. The methods of linkage and methods of linkage quality evaluation should be provided. | Page 6-7 |
| **Results** | | | | | |
| Participants | 13 | (a) Report the numbers of individuals at each stage of the study (*e.g.*, numbers potentially eligible, examined for eligibility, confirmed eligible, included in the study, completing follow-up, and analysed)  (b) Give reasons for non-participation at each stage.  (c) Consider use of a flow diagram | Figure S1 and Figure S2. | RECORD 13.1: Describe in detail the selection of the persons included in the study (*i.e.,* study population selection) including filtering based on data quality, data availability and linkage. The selection of included persons can be described in the text and/or by means of the study flow diagram. | Page 6-7;  Figure S1;  Figure S2. |
| Descriptive data | 14 | (a) Give characteristics of study participants (*e.g.*, demographic, clinical, social) and information on exposures and potential confounders  (b) Indicate the number of participants with missing data for each variable of interest  (c) *Cohort study* - summarise follow-up time (*e.g.*, average and total amount) | Page 10-11;  Table 1  Table S1;  Table S2.  Table S2   Page 10-11; |  |  |
| Outcome data | 15 | *Cohort study* - Report numbers of outcome events or summary measures over time | Page 10-11;  Table 2; Table 3; |  |  |
| Main results | 16 | (a) Give unadjusted estimates and, if applicable, confounder-adjusted estimates and their precision (e.g., 95% confidence interval). Make clear which confounders were adjusted for and why they were included.  (b) Report category boundaries when continuous variables were categorized  (c) If relevant, consider translating estimates of relative risk into absolute risk for a meaningful time period | Page 10-11;  Table 2;  Table 3; |  |  |
| Other analyses | 17 | Report other analyses done—e.g., analyses of subgroups and interactions, and sensitivity analyses | Page 11-12 |  |  |
| **Discussion** | | | | | |
| Key results | 18 | Summarise key results with reference to study objectives | Page 13 |  |  |
| Limitations | 19 | Discuss limitations of the study, taking into account sources of potential bias or imprecision. Discuss both direction and magnitude of any potential bias | Page 13-15 | RECORD 19.1: Discuss the implications of using data that were not created or collected to answer the specific research question(s). Include discussion of misclassification bias, unmeasured confounding, missing data, and changing eligibility over time, as they pertain to the study being reported. | Page 13-14 |
| Interpretation | 20 | Give a cautious overall interpretation of results considering objectives, limitations, multiplicity of analyses, results from similar studies, and other relevant evidence | Page 13-15 |  |  |
| Generalisability | 21 | Discuss the generalisability (external validity) of the study results | Page 13-16 |  |  |
| **Other Information** | | | | | |
| Funding | 22 | Give the source of funding and the role of the funders for the present study and, if applicable, for the original study on which the present article is based | Page 17 |  |  |
| Accessibility of protocol, raw data, and programming code |  | .. | Page 6. | RECORD 22.1: Authors should provide information on how to access any supplemental information such as the study protocol, raw data, or programming code. | Page 6. |

Benchimol EI, Smeeth L, Guttmann A, Harron K, Moher D, Petersen I, Sørensen HT, von Elm E, Langan SM, the RECORD Working Committee. The Reporting of studies Conducted using Observational Routinely collected health Data (RECORD) Statement. https://doi.org/10.1371/journal.pmed.1001885.

Page numbers refer to the original submission.
